# Metabolomic alterations in follicular fluid across female infertility conditions in IVF: a systematic review

**DOI:** 10.1007/s10815-026-03925-y

**Published:** 2026-06-04

**Authors:** Lucca D’Arco Correa, Fernanda Souza Peruzzato, Yara Maria Rauh Muller

**Affiliations:** 1 Sapientiae Institute Association for Assisted Human Reproduction, São Paulo, Brazil; 2 Postgraduate Program in Cell and Developmental Biology, Federal University of Santa Catarina, Florianópolis, Brazil; 3 Laboratory of Animal Reproduction and Development (LRDA), Biological Sciences Center, Federal University of Santa Catarina, Florianópolis, Brazil

**Keywords:** Metabolomics, Follicular fluid, In vitro fertilization, Female infertility

## Abstract

**Purpose:**

This systematic review investigates the relationship between follicular fluid metabolomic profiles and different female infertility conditions, focusing on the molecular alterations associated with each condition.

**Methods:**

A systematic literature review was conducted in accordance with PRISMA guidelines. A comprehensive search was performed in the Embase, MEDLINE, and Cochrane databases. Study selection was carried out using the Rayyan platform based on predefined inclusion and exclusion criteria.

**Results:**

The follicular fluid metabolome appears to be influenced by various infertility-related factors. Key metabolites, including glucose, lactate, lipids (such as diacylglycerols, triacylglycerols, fatty acids, sphingomyelins, and oleic acid), and amino acids (including lysine, valine, glutamate, histidine, and glutamine), were consistently reported as altered across the included studies. These findings suggest their potential relevance for advancing embryology research and clinical assisted reproduction.

**Conclusion:**

Metabolomics, due to its analytical sensitivity and predictive potential, may contribute to improving in vitro fertilization (IVF) laboratory practices and enhancing clinical outcomes.

**Supplementary Information:**

The online version contains supplementary material available at https://doi.org/10.1007/s10815-026-03925-y.

## Introduction

Metabolomics, a term introduced by Oliver Fiehn [1], refers to the comprehensive study of metabolites, small molecules (up to 1500 Da), that are intermediates or end products of metabolism within a biological sample. It enables the identification and quantification of metabolites (e.g., amino acids, carbohydrates, fatty acids, and nucleotides) in biological systems, including fluids, cells, tissues, organs, and whole organisms. This approach improves the understanding of phenotypes, metabolic pathway dynamics, and cellular functions, and facilitates the identification of biomarkers associated with developmental stages, physiological processes, diseases, and specific phenotypes. Metabolomics has been increasingly applied across multiple fields, including clinical analysis, nutrition, sports science, environmental studies, forensic toxicology, pathogen research, and, more recently, assisted human reproduction [2].

In assisted reproductive technology (ART), metabolomics has been applied primarily to identify fertility biomarkers and assess embryo quality [3]. In women, metabolomic analysis of follicular fluid (FF) is the most extensively studied approach in humans, although analysis of embryo culture media has also gained attention. FF is a dynamic component of the ovarian follicle, rich in metabolites [4], that supports communication between the oocyte and surrounding follicular cells and plays a critical role in oocyte development and fertilization competence [5]. Previous studies have investigated how FF metabolites influence oocyte quality, maternal conditions, and embryo development [6]. The identification of relevant biomarkers may support the selection and classification of oocytes based on quality, contribute to the optimization of fertilization and live birth rates, and enable more individualized treatment strategies. Furthermore, a better understanding of follicular development may support the development of personalized embryo culture media.

This study aimed to evaluate the interaction between follicular fluid metabolomic profiles and female infertility conditions through a systematic literature review. Specifically, we investigated whether advanced ovarian age and other female infertility factors are associated with alterations in the metabolomic profile of follicular fluid in women undergoing in vitro fertilization. In addition, potential biomarkers related to infertility and maternal conditions, as well as their association with fertilization and live birth outcomes, were assessed.

## Methods

### Study design and search strategy

This study was conducted as a systematic literature review in accordance with the PRISMA (Preferred Reporting Items for Systematic Reviews and Meta-Analyses) guidelines [7]. A comprehensive search using MeSH (Medical Subject Headings) terms was performed in the Embase, MEDLINE, and Cochrane databases. The completed PRISMA 2020 checklist is provided in Supplement 1.

The following search string was applied consistently across all databases: *(Metabolomics) AND (Follicular fluid) AND (*In vitro* fertilization treatment) AND (Human).* Search results were imported into Rayyan, an online platform that supports systematic review workflows by enabling duplicate removal, title and abstract screening, and structured data organization.

### Study selection

The Rayyan platform was used to manage and screen the retrieved articles. Titles and abstracts were initially screened by one reviewer according to predefined inclusion and exclusion criteria. To ensure consistency, all records initially excluded at this stage (approximately 40% of screened records) were independently reassessed by a second reviewer to verify the appropriateness of exclusions and minimize the risk of inadvertently excluding eligible studies. There was a high level of agreement between reviewers, with only three records requiring attention, of which two were subsequently reclassified for full-text assessment. Discrepancies were resolved through discussion until consensus was reached. Full-text articles were then independently assessed by two reviewers for final inclusion, and any disagreements were resolved by consensus among all reviewers.

Although formal inter-rater agreement statistics were not calculated and full duplicate screening of all records at the title and abstract stage was not performed, verification of all excluded records by a second reviewer, together with predefined criteria and consensus-based decisions, contributed to the consistency of study selection.

This study follows the PICO framework:

- Patients (P): women undergoing metabolomic analysis of follicular fluid
- Intervention (I): ovarian stimulation for IVF due to female infertility
- Comparison (C): women undergoing IVF due to male factor infertility and/or favorable reproductive outcomes or prognosis
- Outcome (O): impact on follicular fluid metabolites

### Inclusion and exclusion criteria

Studies were included if they involved women of reproductive age undergoing assisted reproductive treatment and metabolomic analysis of follicular fluid. Both prospective and retrospective studies, as well as randomized and nonrandomized designs, were considered. The search included studies published between January 2010 and December 2023. Only articles published in English or Portuguese were eligible.

Exclusion criteria included review articles, meta-analyses, opinion papers, and case reports; studies involving women outside reproductive age or using contraceptives; and studies that did not apply metabolomic analysis to follicular fluid.

### Search scope and additional sources

Study selection was based exclusively on records retrieved from the predefined database searches. Reference lists of included studies were reviewed for contextual purposes only and were not used to identify or include additional studies in the systematic analysis. Grey literature was not included in the systematic review; however, selected sources (e.g., theses) were consulted to support the introduction when relevant.

The complete search strategy is provided in Supplement 2.

### Data extraction

The following data were extracted from the included studies: first author, year of publication, country, study design, infertility condition, total number of participants, number of women undergoing IVF, number of controls (when available), mean age and BMI, hormonal and IVF outcome differences, medications and dosages, metabolomic platforms, identified metabolites, potential biomarkers, differentially expressed metabolic pathways, associations between metabolites and IVF outcomes, and main conclusions.

### Quality assessment of studies

The Newcastle‒Ottawa quality assessment score (NOS) [8] was used to evaluate the methodological quality of the included studies. This tool consists of eight items assessing cohort and case–control studies across three dimensions: selection, comparability, and outcome/exposure (outcome for cohort studies and exposure for case–control studies). Each item has response options indicating high methodological quality, assigned as stars. The selection and exposure/outcome dimensions contain four and three items, respectively, each earning up to one star, whereas comparability has one item that can receive up to two stars. The total score ranges from zero to nine, enabling a semiquantitative evaluation where higher-quality studies receive more stars.

### Protocol registration

This systematic review protocol was registered in the Open Science Framework (OSF). As the study had already been completed at the time of registration, this represents a retrospective registration aimed at ensuring transparency and adherence to open science practices. All methodological steps and eligibility criteria were defined prior to data extraction and consistently followed throughout the review.

## Results

**Fig. 1:**
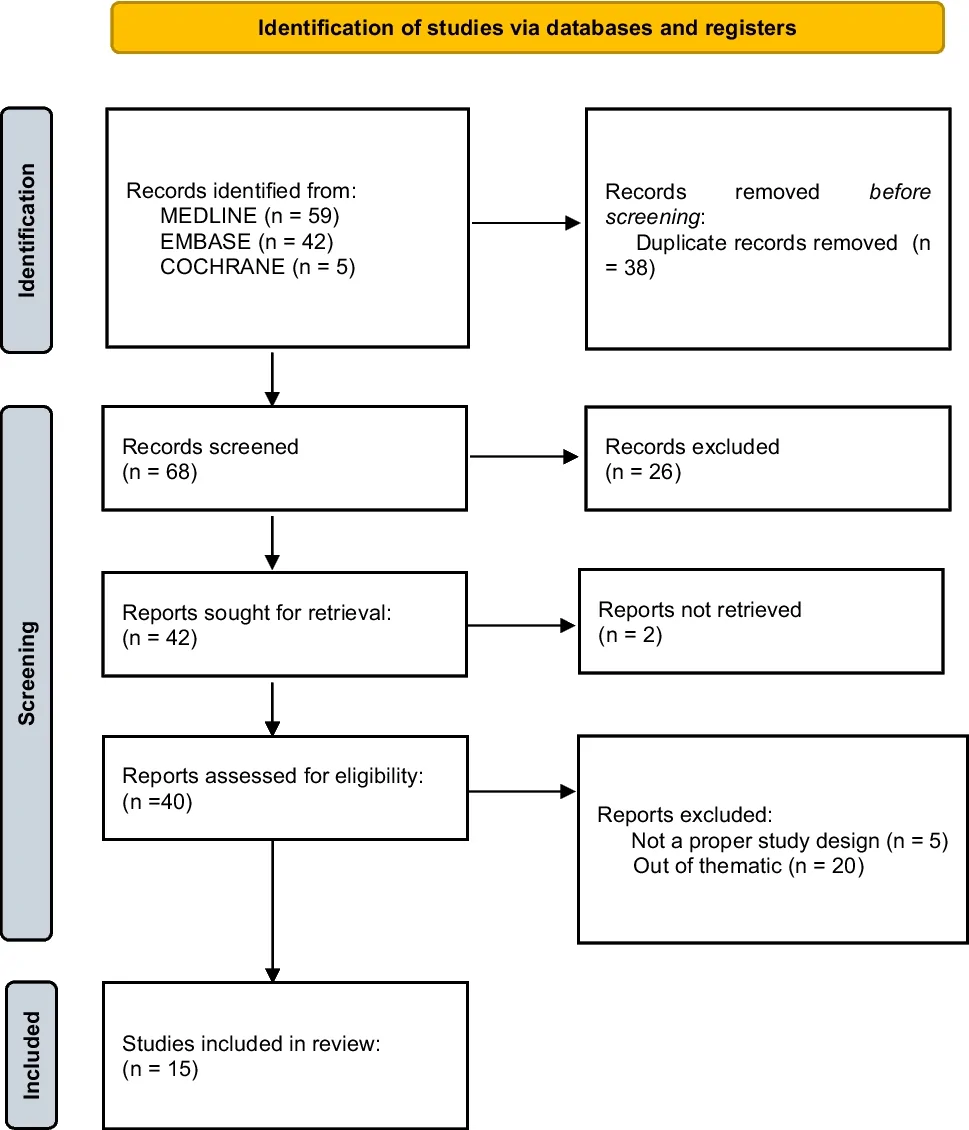
PRISMA flow diagram for selection and inclusion of studies for review. Source: Page et al., 2021 [7]

A total of 15 studies met the inclusion criteria and were included in this review. The study selection process is summarized in the PRISMA flow diagram (Fig. 1).

The included studies were published between October 2014 and October 2023 and were conducted in China, Brazil, Turkey, and Italy. Overall, 1133 women of reproductive age undergoing IVF were included and categorized into control and study groups. The main characteristics of the included studies are presented in Table 1, with additional details provided in Supplement 3 and Table 2.

**Table 1:** Characteristics of the articles addressing the impacts of female infertility conditions on the metabolomics of follicular fluid

*Author*	*Year*	*Country*	*Study design*	*Participants*	*Study group (n)*	*Control group (n)*	*Age (years)—study group (mean* ± *SD)*	*Age (years)—control group (mean* ± *SD)*	*BMI (kg/m*^*2*^*) –study group (mean* ± *SD)*	*BMI (kg/m*^*2*^*)—control group (mean* ± *SD)*
*Xia L.*et al*.* [17]	Sep-2014	China	Case–control study	28	13 (recurrent implantation failure)	15 (positive pregnancy in first IVF cycle)	34.0 ± 3.9	33.3 ± 3.2	NR	NR
*Cordeiro F.*et al*.* [10]	Oct-2015	Brazil	Case–control study	20	10 (stage III or IV endometriosis with endometrioma)	10 (tubal factor or mild male factor)	32.3 ± 2.67	34.3 ± 3.3	23.30 ± 2.57	24.06 ± 2.03
*Cordeiro F. *et al [21]	Mar-2018	Brazil	Prospective case–control study	81	PCOS group (*n* = 15); high responder group (*n* = 44)	22 normo responder women	PCOS 30.6 ± 3.31; HR 31.5 ± 3.83	33.95 ± 3.15	PCOS 26.05 ± 5.52; HR 23.13 ± 2.77	23.35 ± 3.86
*Karaer A.*et al*.* [11]	Apr-2018	Turkey	Prospective case–control study	24	12 (ovarian endometriosis)	12 (pure male factor)	33.3 ± 5.28	31.0 ± 5.21	25.1 ± 2.62	24.6 ± 3.53
*Morelli M. *et al*.* [4]	Jan-2019	Italy	Case–control study	53	43 (13–unexplained infertility; 10 tubal factor; 8 endometriosis; 12 PCOS)	10 (mild or moderate male factor)	Unexplained infertility 36.8 ± 3.5; tubal factor 36.7 ± 4.2; endometriosis 38.6 ± 3.0, PCOS 34.7 ± 4.6	36.0 ± 4.0	Unexplained infertility 22.3 ± 3.1; tubal factor 24.2 ± 2.8; endometriosis 25.1 ± 4.0, PCOS 24.8 ± 5.3	22.5 ± 3.4
*Song J. *et al*.* [18]	Jul-2019	China	Case–control study	44	22 (history of recurrent spontaneous abortion and tubal factor)	22 (history of induced abortion from a normal pregnancy and tubal factor)	29.7 ± 3.8	30.3 ± 3.5	21.6 ± 1.2	21.7 ± 1.1
*Dogan B. *et al*.* [15]	Jan-2020	Turkey	Case–control study	54	23 (advanced maternal age > 40 years)	31 (normal ovarian reserve)	41.3 ± 1.6	30.4 ± 2.5	26.1 ± 3.1	24.4 ± 2.6
*Zhang X. *et al*.* [16]	May-2020	China	Cohort study	230	Advanced age group (*n* = 90)	Younger age group (*n* = 140)	40.51 ± 3.47	29.38 ± 3.01	22.79 ± 1.34	21.62 ± 6.67
*Lazzarino G. *et al*.* [9]	Aug-2021	Italy	Case–control study	180	145 (Endometriosis; age-related reduced ovarian reserve; reduced ovarian reserve; unexplained infertility; genetic infertility)	35 (pure male factor)	32.7 ± 7.6	30.6 ± 4.7	24.6 ± 5.9	23.4 ± 5.1
*Guan S. *et al*.* [20]	Sep-2022	China	Case–control study	60	PCOS group (*n* = 30)	Tubal factor group (*n* = 30)	28.88 ± 3.91	27.78 ± 2.65	23.58 ± 3.83	22.71 ± 2.6
*Shen H. *et al*.* [19]	Oct-2022	China	Cohort study	84	28 (PCOS); 28 (diminished ovarian reserve)	28 (normo-ovulatory women)	PCOS: 29.68 ± 3.88; DOR: 33.93 ± 3.11	32.25 ± 3.58	PCOS: 23.5 ± 3.94; DOR: 23.29 ± 3.75	22.78 ± 2.59
*Dabaja M. *et al [12]	Nov-2022	Brazil	Case–control study	49	25 (unexplained infertility, tuboperitoneal factor, uterine factor, endometriosis/negative biochemical pregnancy)	24 (unexplained infertility, tuboperitoneal factor, uterine factor, endometriosis/positive biochemical pregnancy)	< 37	< 37	18.5–30	18.5–30
*Sun Y. *et al*.* [22]	Jan-2023	China	Case–control study	87	PCOS–normal ovarian response (21); PCOS–OHSS (24)	Normal ovarian response (27); OHSS (15)	20–40	20–40	20–30	20–30
*Li J. *et al*.* [13]	Feb-2023	China	Prospective clinical study	54	26 (diminished ovarian reserve)	28 (normal ovarian reserve)	34.04 ± 3.8	30.29 ± 2.62	21.57 ± 1.43	20.48 ± 1.74
*Liu A. *et al*.* [14]	Oct-2023	China	Case–control study	85	50 (diminished ovarian reserve)	35 (normal ovarian reserve)	31.28 ± 2.63	29.83 ± 2.94	21.45 ± 2.32	20.73 ± 1.81

*NR, not reported by the original study

*Abbreviations: *OHSS*, ovarian hyperstimulation syndrome; *PCOS*, polycystic ovary syndrome

**Table 2:** Summary of studies that addressed the impacts of female infertility conditions on follicular fluid

*Author*	*Protocol*	*Difference between groups*	*Female infertility conditions*	*Metabolomic platforms*	*Metabolites*	*Metabolic pathways*	*Biomarker*	*Correlation*	*Conclusion*
**Cordeiro F. et al. **[10]	FSH (225 IU/day, starting on D2); GnRH antagonist (Cetrotide, 0.25 mg/day, when the dominant follicle reached 13 mm); hCG (250 mg, 35 h before retrieval when the dominant follicle reached 17 mm)	No differences	**III/IV endometriosis**	ESI-QTOF, MS/MS	Phosphatidylglycerol phosphate, phosphatidylcholine, phosphatidylserine, phosphatidylinositol bisphosphate, sphingolipids, phosphatidylcholines	NA	Phosphatidylglycerol phosphate, phosphatidylcholine, phosphatidylserine, phosphatidylinositol bisphosphate, sphingolipids, phosphatidylcholines	NA	The results show an effective approach for follicular fluid collection from women with endometriosis and ovarian endometrioma. Many of the identified lipids showed differential expression between groups and are involved in follicular development during IVF and the cleavage process, as well as apoptosis and cell proliferation
**Karaer A. et al. **[11]	GnRH antagonist (D6 of cycle); recombinant FSH (D2 or D3); hCG (10,000 IU, 36 h before retrieval when at least three follicles reached 18 mm)	No differences	**Ovarian endometriosis**	NMR spectroscopy	Lactate, β-glucose, pyruvate, valine	NA	NA	NA	The study reveals that metabolites with altered concentrations were identified in patients with ovarian endometriosis compared with healthy women, highlighting the role of follicular fluid in energy supply
**Li J. et al. **[13]	GnRH antagonist or PPOS protocols with gonadotropins and hCG	Lower mature oocytes, fertilization, and good-quality embryos in study group	**Diminished ovarian reserve**	LC–MS timsTOF	Amino acids, nucleosides, indoles, steroids, phospholipids	Aminoacyl-tRNA biosynthesis, tryptophan metabolism, purine metabolism	NA	Positive correlation with AMH, AFC, oocytes, and fertilization	Results support the study of DOR pathogenesis and the search for new diagnostic markers
**Liu A. et al** [14]	Short- or long-term GnRH agonist; PPOS	Older age and higher basal FSH in study group; lower AMH, AFC, oocytes, embryos	**Diminished ovarian reserve**	LC–MS/MS	Indoles and tryptophan derivatives (reduced)	NA	NA	Tryptophan correlated with viable embryo rate	Findings suggest tryptophan and indole metabolites may have antioxidant capacity in follicular fluid and reflect gut–follicle microenvironment interactions
**Shen H. et al. **[19]	GnRH antagonis; PPOS	Higher LH, AMH, and number of retrieved oocytes in PCOS; higher FSH in diminished ovarian reserve	**PCOS and diminished ovarian reserve**	UPLC -MS/MS	Steroid metabolites, DG, PE, nucleosides, sphingolipids, amino acid derivatives	Choline metabolism, steroid hormone biosynthesis, sphingolipid signaling, purine and pyrimidine metabolism	Hydroxyestrone sulfate, pregnanediol-3-glucuronide	Acetylcarnitine negatively correlated with FSH; phosphatidylcholine positively correlated with AMH and transferable embryo rate	Metabolic changes were examined in non-PCOS and diminished ovarian reserve patients, providing insights into follicular microenvironment alterations in recurrent implantation failure
**Dogan B. et al. **[15]	Gonadotropin (from cycle day 2); GnRH antagonist (from day 5 or 6); r-hCG (250 µg, 34–36 h before oocyte retrieval when ≥ 3 follicles reached 18 mm)	Higher in study group: total gonadotropin dose. Lower in study group: number of oocytes, mature oocytes, and embryos	**Advanced maternal age**	NMR spectroscopy	Lower in study group: α-glucose and β-glucose. Higher in study group: lactate and trimethylamine N-oxide	NA	NA	NA	Analyses showed that the follicular fluid metabolomic profile of women of advanced maternal age demonstrates differences in glucose and lactate metabolism, which may be associated with dysregulated follicular metabolism
**Zhang X. et al. **[16]	r-FSH and hMG (300 IU/day for 4 days); GnRH antagonist (0.25 mg/day when dominant follicle reached 13–14 mm); hCG or r-hCG	Mean age, basal FSH, LH, and E2. Lower antral follicle count in advanced ovarian age group. Oocyte recovery rate, 2PN cleavage rate, and transferable embryo rate decrease with age	**Advanced maternal age**	UPLC-TOF	4,5-Dihydroorotic acid, maleylacetoacetic acid, 4-oxo-retinoic acid, arachidonate, nicotine glucuronide, DG, TG, deoxycorticosterone, LysoPC, phytosphingosine, phosphatidylcholine (16:0/22:0), 5,6-dihydrouridine	Pyrimidine metabolism, aromatic nucleus metabolism, vitamin metabolism, lipid metabolism, glucose metabolism, mineralocorticoid biosynthesis, nucleic acid metabolism	NA	NA	Significant changes in lipid metabolism were observed, mainly due to increased arachidonic acid expression, affecting oocyte development, while LPC participates in follicular development regulation
**Xia L. et al. **[17]	GnRH agonist (daily, starting in the mid-luteal phase of the previous menstrual cycle); u-FSH or r-FSH (daily); hCG (36–40 h before oocyte retrieval, when at least two follicles reached 18–20 mm)	Duration of infertility and number of previous cycles	**Recurrent implantation failure**	GC–MS	α-Aminoadipic acid, 6-hydroxy-2-aminohexanoic acid, aminomalonic acid, L-aspartic acid, 3-hydroxybutyric acid, cholesterol, glutaric acid, succinic acid, 3-methyl-2-ketobutyric acid, L-valine, L-threonine, L-isoleucine, L-cysteine, L-serine, L-proline, L-alanine, L-phenylalanine, L-lysine, L-methionine, L-ornithine	NA	NA	NA	The study raises the possibility that metabolomic profiling may be a realistic way to optimize embryo selection to achieve successful pregnancy, with potential advantages over morphological selection
**Song J. et al. **[18]	GnRH agonist (Decapeptyl, 0.05 mg/day – starting in the mid-luteal phase of the previous menstrual cycle)	Only in the number of spontaneous abortions	**Recurrent spontaneous abortion**	UPLC-TOF and UPLC-QTRAP	Dehydroepiandrosterone, lysoPC, phenylalanine, linoleate, oleic acid, docosahexaenoic acid, lithocholic acid, 25-hydroxyvitamin D3, 25-hydroxycholesterol, 13-hydroxy-α-tocopherol, leucine, tryptophan	Aminoacyl-tRNA biosynthesis; phenylalanine, tyrosine and tryptophan biosynthesis; nitrogen metabolism; linolenic acid metabolism; steroid hormone biosynthesis; fatty acid biosynthesis	Dehydroepiandrosterone, lysoPC, phenylalanine, linoleate, oleic acid, docosahexaenoic acid, lithocholic acid, 25-hydroxyvitamin D3, 25-hydroxycholesterol, 13-hydroxy-α-tocopherol, leucine, tryptophan	NA	The study suggests that the metabolomic profile has great potential to differentiate patients with recurrent miscarriage from those without this condition
**Guan S. et al. **[20]	GnRH antagonist	Higher basal LH, testosterone, and infertility duration in PCOS group	**PCOS**	UPLC -MS/MS	DG, androsterone sulfate, L-erythrulose, LysoPE, palmitoylcarnitine, linoleyl carnitine, LysoPA	Glycerolipid metabolism, glycerophospholipid metabolism, arginine and proline metabolism, drug metabolism (cytochrome P450)	LysoPE, DG, linoleyl carnitine, androsterone sulfate, LysoPA	NA	Differential metabolites are related to multiple metabolic pathways, with glycerophospholipid metabolism identified in both sources, indicating differential lipid metabolism in early embryo development
**Sun Y. et al. **[22]	Long protocol with GnRH agonist	Higher E2, follicle number, retrieved and mature oocytes in OHSS	**OHSS and PCOS**	GC–MS	Unsaturated fatty acids, organic acids, amino acids	NA	Fatty acids, organic acids, amino acids	Positive correlation with follicle number, oocytes, mature oocytes, and E2	Findings suggest metabolic abnormalities in follicular fluid are associated with OHSS development, with shared etiological factors between PCOS and OHSS
**Cordeiro F. et al. **[21]	r-FSH (Gonal-F, 225 IU/day, starting on D2); GnRH antagonist (Cetrotide, 250 µg/day, when the dominant follicle reached 13 mm); hCG (Ovidrel, 250 mg, 35 h before retrieval when the dominant follicle reached 17 mm)	Control group patients had higher mean age, FSH levels, FSH/LH ratio, and number of transferred embryos. PCOS and HR groups showed higher mean numbers of aspirated follicles, MII and MI follicles, injected oocytes, fertilized oocytes, embryos on days 2 and 3 of culture, poorer-quality embryos on day 2, and good- or poor-quality embryos on day 3. Oocyte recovery rate was lower in PCOS than HR. HR had a higher number of good-quality oocytes on day 3 and lower implantation rates in the study groups compared with controls	**PCOS/high responders**	UPLC -MS	Control group: higher levels of fatty acids, diacylglycerol, triacylglycerol, ceramide, ceramide-phosphate, phosphatidylcholine, and sphingomyelin PCOS group: higher abundancy of molecule of m/z 144.0023 (not attributed class)	Arachidonic acid metabolism, glycerophospholipid metabolism, glycosphingolipid metabolism, linoleate metabolism	NA	NA	Considering that the control group was composed of patients that achieved pregnancy after IVF treatment, it was possible to compare follicular environment that is suitable for proper oocyte development and further pregnancy outcome with another that is affected by hyper response due to PCOS or its secondary characteristics
**Lazzarino G. et al. **[9]	A) GnRH antagonist + menotropin from day 2 + r-hCG; B) GnRH agonist from day 1 + menotropin from day 2 + r-hCG	Infertile group showed lower number of retrieved oocytes, mature oocytes, fertilized oocytes, blastocysts, good-quality blastocysts, and positive β-hCG	**Multiple causes of female infertility (PCOS, endometriosis and advanced maternal age)**	HPLC	Glucose, lactate, hypoxanthine, β-pseudouridine, uracil, cytosine, cytidine, GSH, ascorbic acid, MDA, 8-OH-dG, nitrite, nitrate, trans-retinol, 25-OH-cholecalciferol, α-tocopherol, coenzyme Q10, carotenoids, amino acids	Energy metabolism, antioxidant defense, oxidative and nitrosative stress biomarkers, free amino acids	Glucose, lactate, hypoxanthine, β-pseudouridine, uracil, cytosine, cytidine, GSH, ascorbic acid, MDA, 8-OH-dG, nitrite, nitrate, trans-retinol, 25-OH-cholecalciferol, α-tocopherol, coenzyme Q10, carotenoids, amino acids	NA	Female infertility is characterized by abnormalities in the concentrations of 27 follicular-fluid compounds, reflecting alterations in energy metabolism, oxidative/nitrosative stress biomarkers, and free amino acids
**Dabaja M. et al. **[12]	Low-cost protocol (100 IU of rFSH), antagonist, hCG	NA	**Multiple causes of female infertility (endometriosis, uterine factors, tubal factors and idiopathic factors)**	ESI-LTQ-XL Orbitrap	PA, GlcCer, PE, PC, PG, PI, vitamin D3 derivatives	Cell signaling, proliferation, inflammation, oncogenesis, apoptosis	NA	NA	Findings suggest follicular fluid can identify biomarkers negatively affecting pregnancy in female infertility
**Morelli M. et al. **[4]	Recombinant or urinary FSH, GnRH antagonist	Median number of follicles monitored, total number of follicles retrieved, mature follicles, pregnancy rate	**Multiple causes of female infertility (unexplained infertility, tubal factor, endometriosis, PCOS)**	NMR spectroscopy	Acetate, β-hydroxybutyrate, leucine, threonine, lactate, glucose, creatine, glycerol, citrate, valine, and unsaturated lipids	NA	acetate, β-hydroxybutyrate, leucine, threonine, lactate, glucose, creatine, glycerol, citrate, valine, and unsaturated lipids	Positive correlation: BMI—alanine and aspartate; number of follicles monitored—choline, creatine, glucose, and glycerol; number of follicles retrieved—aspartate, glucose, and glutamate; number of mature oocytes—choline, creatine, and glucose; number of embryos produced by mature oocytes—citrate, glucose, and valine. Negative correlation: number of oocytes retrieved, acetate; β-hCG produced during pregnancy, lactate; maternal age, number of follicles, increased lactate, and decreased aspartate and glucose	Metabolic profile analysis allows for the identification of follicle dysfunction in women with PCOS and endometriosis, and the identification of specific biomarkers suggests an abnormal metabolism of carbohydrates, lipids, and amino acids

*NA, not applicable (e.g., when biomarkers, metabolic pathways, or correlations were not investigated or explicitly defined)

*Abbreviations: *AFC*, antral follicle count; *DG*, dominant follicle group; *DOR*, diminished ovarian reserve; *ESI-LTQ-XL*, electrospray ionization linear ion trap mass spectrometry (LTQ XL); *ESI-QTOF-MS*, electrospray ionization quadrupole time-of-flight mass spectrometry; *FSH*, follicle-stimulating hormone; *GC–MS*, gas chromatography–mass spectrometry; *GnRH*, gonadotropin-releasing hormone; *hCG*, human chorionic gonadotropin; *LC–MS timsTOF*, liquid chromatography–mass spectrometry with trapped ion mobility time-of-flight; *MS/MS*, tandem mass spectrometry; *NMR*, nuclear magnetic resonance; *OHSS*, ovarian hyperstimulation syndrome; *PCOS*, polycystic ovary syndrome; *PE*, phosphatidylethanolamine; *PPOS*, progestin-primed ovarian stimulation; *UPLC*, ultra-performance liquid chromatography; *UPLC-QTRAP*, ultra-performance liquid chromatography coupled with triple quadrupole linear ion trap mass spectrometry

The studies evaluated women with different infertility conditions and investigated how these conditions influence the metabolomic profile of follicular fluid. Infertility conditions such as endometriosis, polycystic ovary syndrome (PCOS), diminished ovarian reserve, advanced ovarian age, repeated implantation failure, and recurrent miscarriage were addressed. Differences in IVF outcomes, hormonal parameters, and correlations between differentially expressed metabolites and outcomes are summarized in Table 2.

Due to substantial heterogeneity among the included studies, a quantitative synthesis was not considered appropriate. Therefore, findings were analyzed qualitatively and organized according to infertility condition and analytical platform to facilitate interpretation (Table 3).

**Table 3:** Metabolomic findings organized by infertility condition and analytical platform

*Platform*	*Metabolites*	*Key interpretation*
**Endometriosis**		
LC–MS (ESI-QTOF, MS/MS)	Phosphatidylglycerol phosphate, phosphatidylcholine, phosphatidylserine, phosphatidylinositol bisphosphate, sphingolipids	Differential lipid expression associated with follicular development, cleavage, apoptosis, and cell proliferation
NMR	Lactate, β-glucose, pyruvate, valine	Altered energy metabolism, suggesting changes in energy supply within follicular fluid
**Diminished ovarian reserve (DOR)**		
LC–MS timsTOF	Amino acids, nucleosides, indoles, steroids, phospholipids	Altered amino acid and nucleotide metabolism linked to DOR pathophysiology
LC–MS/MS	Reduced indoles and tryptophan derivatives	Possible oxidative stress and metabolic impairment
**Polycystic ovary syndrome (PCOS)**		
UPLC-MS	Fatty acids, diacylglycerol, triacylglycerol, ceramide, phosphatidylcholine, sphingomyelin	Altered lipid metabolism associated with ovarian hyper-response and follicular environment changes
UPLC-MS/MS	DG, androsterone sulfate, LysoPE, palmitoylcarnitine, linoleyl carnitine, LysoPA	Differential lipid metabolism and involvement of glycerophospholipid pathways in early embryo development
GC–MS	Unsaturated fatty acids, organic acids, amino acids	Metabolic abnormalities associated with OHSS, suggesting shared mechanisms with PCOS
**Advanced maternal age**		
NMR	↓ α-glucose, ↓ β-glucose, ↑ lactate, trimethylamine N-oxide	Altered glucose metabolism and metabolic dysregulation in follicular fluid
UPLC-TOF	Arachidonate, DG, TG, LysoPC, phosphatidylcholine, phytosphingosine	Altered lipid metabolism affecting oocyte development
**Recurrent implantation failure (RIF)**		
GC–MS	↑ Amino acids (valine, leucine, threonine, etc.), ↓ organic acids, cholesterol	Distinct metabolic profiling may improve embryo selection beyond morphology
**Recurrent miscarriage**		
UPLC-TOF/UPLC-QTRAP	Dehydroepiandrosterone, lysoPC, phenylalanine, linoleate, DHA, tryptophan	Distinct metabolic profile potentially useful for differentiating patients

Abbreviations: *DHA*, docosahexaenoic acid; *DG*, diacylglycerol; *DOR*, diminished ovarian reserve; *ESI*, electrospray ionization; *GC–MS*, gas chromatography–mass spectrometry; *LC–MS*, liquid chromatography–mass spectrometry; *LysoPA*, lysophosphatidic acid; *LysoPC*, lysophosphatidylcholine; *LysoPE*, lysophosphatidylethanolamine; *MS/MS*, tandem mass spectrometry; *NMR*, nuclear magnetic resonance; *OHSS*, ovarian hyperstimulation syndrome; *PCOS*, polycystic ovary syndrome; *QTOF*, quadrupole time-of-flight; *RIF*, recurrent implantation failure; *TG*, triacylglycerol; *timsTOF*, trapped ion mobility spectrometry time-of-flight; *UPLC-MS*, ultra-performance liquid chromatography–mass spectrometry; *UPLC-MS/MS*, ultra-performance liquid chromatography–tandem mass spectrometry; *UPLC-QTRAP*, ultra-performance liquid chromatography coupled to QTRAP mass spectrometer; *UPLC-TOF*, ultra-performance liquid chromatography–time-of-flight

Metabolomic findings varied according to both infertility condition and analytical platform. Studies on PCOS predominantly reported alterations in lipid metabolism, particularly involving phospholipids and fatty acids, mainly identified through LC–MS-based approaches. In contrast, studies investigating advanced maternal age and ovarian endometriosis more frequently reported alterations in energy metabolism, including glucose and lactate, particularly in NMR-based analyses.

Studies focusing on endometriosis also demonstrated alterations in lipid and inflammation-related metabolites, although findings varied depending on the analytical platform employed. These differences highlight the combined influence of biological condition and methodological approach on metabolomic outcomes.

Across the included studies, several metabolites were consistently reported as differentially expressed, including lactate, glucose, pyruvate, valine, lipids (particularly fatty acids), glutamate, cholesterol, nucleosides, and other amino acids and carbohydrates. These metabolites were associated with multiple metabolic pathways, including amino acid metabolism, steroidogenesis, sphingolipid signaling, folate biosynthesis, nicotinate metabolism, coenzyme A (CoA) and pantothenate biosynthesis, aminoacyl-tRNA biosynthesis, nitrogen metabolism, and fatty acid and linolenic acid biosynthesis.

**Fig. 2:**
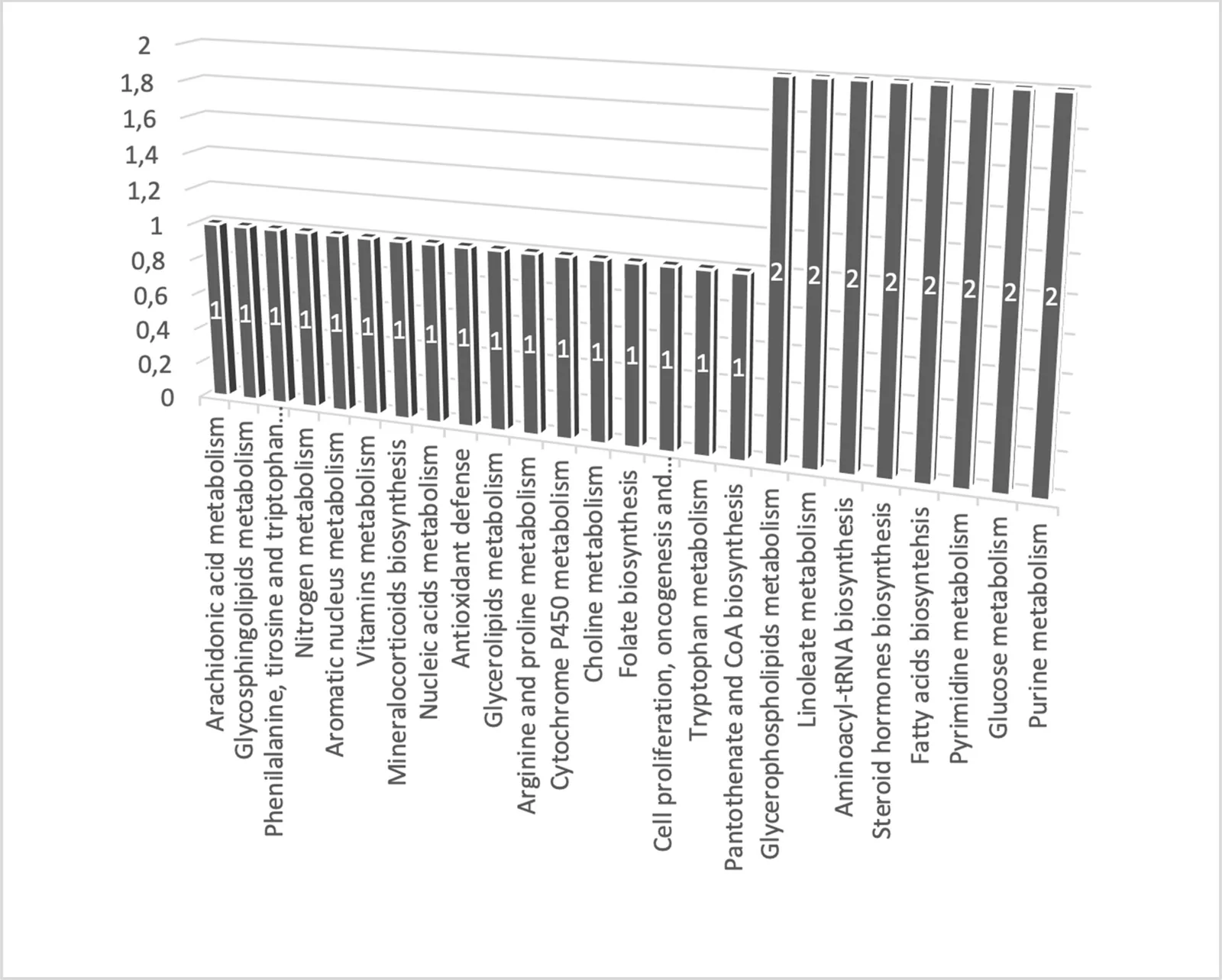
Differentially expressed metabolic pathways and number of studies that identified the same pathway

Among the 15 studies, a total of 25 differentially expressed metabolic pathways were identified in follicular fluid. Eight pathways were reported in two studies, whereas the remaining 17 were identified in a single study. These pathways are represented in Fig. 2.

### Newcastle‒Ottawa methodological quality assessment

The assessment of the methodological quality of each study via the Newcastle‒Ottawa Scale is presented in Supplement 4. Among the 15 included articles, scores ranged from 7–9, with an average of 7.87 stars, indicating high methodological quality. Studies scoring 7–9 points are considered high quality, 5–6 points moderate quality, and ≤ 4 points low quality. Given that no studies were classified as low quality, sensitivity analyses excluding lower-quality studies were not performed. The lowest score in this review (7) was assigned to studies that lacked a control group for comparability. A commonly reported limitation across studies was the limited sample representativeness, which may affect the robustness of the findings. Additionally, variability in patient characteristics may limit the ability to standardize participant selection. Future studies should aim to include larger and more homogeneous cohorts.

## Discussion

Metabolomic analysis of follicular fluid in patients undergoing IVF provides valuable insights into biological processes associated with reproductive outcomes. The identification of infertility-related metabolic alterations, as well as relevant metabolites to oocyte and embryo development, represents a potential advance in reproductive medicine. From both clinical and laboratory perspectives, this approach may support improved diagnostic accuracy and contribute to more individualized treatment strategies. A previous study demonstrated the ability to distinguish fertile from infertile women and reported inverse correlations with oocyte yield, embryo development, blastocyst quality, clinical pregnancy, and live birth outcomes, highlighting the importance of follicular fluid metabolic composition in IVF success [9]. Although the role of oocyte factors in embryo development is well established, the molecular determinants of oocyte competence remain incompletely understood. In this context, the follicular fluid metabolome may help to characterize these determinants and support clinical decision-making.

### Infertility conditions

One of the main applications of follicular fluid metabolomics identified in this review is the potential characterization of diseases and conditions associated with female infertility, including endometriosis, diminished ovarian reserve, advanced ovarian age, PCOS, recurrent implantation failure, and recurrent miscarriage. These findings may contribute to the development of more tailored treatment strategies and, potentially, to the optimization of culture conditions.

Cordeiro et al. [10] evaluated 20 women undergoing IVF, including 10 with stage III–IV ovarian endometriosis and 10 controls with tubal or isolated male factor infertility, and identified differential lipid expression in follicular fluid. These lipids are involved in processes such as follicular development, embryo cleavage, apoptosis, and cell proliferation, consistent with the dysregulated cellular behavior observed in endometriosis. Similarly, Karaer et al. [11], also focusing on patients with ovarian endometriosis, reported differential expression of protein and carbohydrate metabolites, suggesting a distortion in the energy supply provided to oocyte by follicular fluid. Another study demonstrated that in women with endometriosis, metabolomic profiling of follicular fluid revealed a clear separation between infertile and pregnant patients, with infertile women showing higher levels of phosphatidic acids, suggesting that dysregulation of lipid signaling pathways related to inflammation, cell proliferation, and aberrant cellular survival may contribute to impaired reproductive outcomes in this subgroup [12].

Furthermore, changes in the metabolome of FF in patients with decreased ovarian reserve were identified. The concentrations of amino acids, indoles, nucleosides, organic acids, steroids, phospholipids, fatty acids, and organic compounds are reduced or increased, which allows the study of the pathogenesis of this syndrome and the identification of differential results in IVF outcomes [13]. These findings may provide more support for treating such patients with a more effective protocol that optimizes oocyte uptake and balances the antioxidant capacity in the follicular microenvironment [14] on the basis of future studies.

Advanced ovarian age, a major contributor to female infertility, was also associated with metabolic alterations. Studies by Dogan et al. [15] and Zhang et al. [16] reported changes in glucose and lactate metabolism, as well as in lipid composition, alongside differences in hormonal profiles and IVF outcomes. These findings suggest that aging is associated with dysregulation of the follicular metabolic environment.

Repeated implantation failure and recurrent spontaneous abortion are two factors that also affect female infertility and may be associated with poor oocyte quality and its environment. Xia et al. [17] evaluated 13 women with repeated implantation failures that had their FF compared to FF from 15 women with positive pregnancies in the first IVF cycle, and differential expression of amino acids and lipids was observed, indicating that such failures may be related to an imbalance in FF and a lower-quality oocyte. Thus, metabolomic profiling has become a realistic way to optimize embryo selection, with potential advantages over morphological selection [17]. Similarly, in cases of recurrent miscarriages, differences in amino acid and lipid profiles were also identified, suggesting that metabolome is capable of differentiating follicles that resulted in miscarriages from those that did not. This approach can be used to better understand the pathogenesis of the disease and, in the future, to diagnose the best oocytes earlier [18]. Thus, metabolomics can play an important role in oocyte selection and prevent unfavorable outcomes during treatment.

Polycystic ovary syndrome was the syndrome with the highest number of selected studies. Evidences suggest that PCOS alters the follicular microenvironment, IVF outcomes, and characteristics of the female organism. The changes caused by this condition may be possible therapeutic and diagnostic targets, which, when identified and treated, contribute to a positive outcome for both the patient’s quality of life and a higher pregnancy and live birth rate [19]. In one study, major alterations in the glycerophospholipid metabolism pathway and other differentially expressed lipids were identified [20], which once again demonstrated the impact of the lipid profile on clinical outcomes related to infertility. Furthermore, two articles reported a relationship between PCOS and ovarian hyperstimulation syndrome. Cordeiro et al. [21] reported that PCOS and hyper response to ovarian stimulation affect the composition of FF, causing a decrease in the concentration of fatty acids, diacylglycerols, triacylglycerols, phosphatidylcholine, sphingomyelin, and ceramides in relation to the control group (normal-responding women who achieved pregnancy after treatment), concluding that the metabolic profile of the hyper response is intermediate between the control profile and the profile of patients with PCOS. However, the results indicated that the hyper response can lead to changes in the presence or absence of polycystic ovary syndrome, which suggests an independent action and collaborates with studies that analyzed the impact of stimulation on the follicular microenvironment. Similarly, the study by Sun et al. [22] revealed nine lipid metabolites that can determine an association between the risk of developing OHSS and patients with PCOS, a finding that can be of great use in the clinic to avoid a hyper response and that strengthens the proposal that the OHSS profile is intermediate between control and PCOS, since such biomarkers are present in both conditions, although the development of OHSS can occur in patients without polycystic ovary syndrome. Furthermore, Morelli et al. demonstrated that women with PCOS exhibit marked alterations in carbohydrate, lipid, and amino acid metabolism. Their findings revealed significantly decreased levels of acetate, β-hydroxybutyrate, leucine, threonine, and lactate, alongside increased concentrations of glucose, creatine, and glycerol in follicular fluid [4].

### Fertility biomarkers

The search for fertility biomarkers is still subjective, owing to limitations in technical knowledge, multiple causes of infertility, and heterogeneous populations, requiring more time and development of analysis tools. However, metabolites such as glucose, lactate, some lipids (diacylglycerols, triacylglycerols, fatty acids, sphingomyelins, and oleic acid, among others), and amino acids (lysine, valine, glutamate, histidine, glutamine, and other amino acids present) can assume this function and be pillars for advances in the areas of embryology and clinically assisted reproduction, since they were altered in most of the selected studies. Therefore, advances in studies are essential to achieve personalized medicine and better success rates [9, 12].

### IVF outcomes

The study of the follicular fluid metabolome in women undergoing IVF has become increasingly important for advancing technologies in assisted reproduction. This approach supports the shift toward personalized treatment strategies by enabling a deeper understanding of the follicular microenvironment and its dynamic processes, ultimately aiming to more closely replicate the physiological conditions of the female reproductive system [4]. Dabaja et al. identified distinct metabolic signatures associated with infertility across different clinical conditions, highlighting that disease-specific lipid and vitamin-related metabolic disruptions are potentially linked to impaired reproductive outcomes [12]. However, accurately defining the specific metabolomic profiles associated with each infertility factor and their precise impact remains challenging. Addressing this gap will be critical for future treatment optimization, particularly with the integration of artificial intelligence. In this context, metabolomics holds substantial potential to improve predictive models and enhance IVF outcomes at the laboratory level, although its clinical applicability still requires further investigation.

### Limitations

The studies included in this review are, in the majority, single-center retrospective studies (case–control), which do not monitor the development of patients on the basis of interference in treatment but only evaluate the results, in addition to the characteristics presented. To further demonstrate the effectiveness of metabolomic analysis of follicular fluid and its applications, prospective studies are needed, if possible, at multiple centers, as a way to validate the discussions and conclusions of these studies and, thus, effectively impact the routine of the reproductive centers and the treatment of patients.

Although most studies reported key demographic variables such as age and BMI, one study did not provide BMI data [17]. Given the role of BMI as a potential confounder in metabolomic analyses, this should be considered when interpreting findings; however, its overall impact is likely limited.

All included studies reported detailed experimental protocols, encompassing ovarian stimulation regimens, follicular fluid collection procedures, and metabolomic analytical methodologies. Nevertheless, the considerable methodological heterogeneity across studies poses challenges for reproducibility and may hinder the interpretation and validation of metabolomic findings at the biomarker level.

Variations in sample collection timing, storage conditions, and analytical platforms between the selected studies may introduce systematic biases. The timing of sample collection relative to follicular development and oocyte retrieval may influence metabolite composition, as dynamic metabolic changes occur throughout folliculogenesis. For example, it has already been reported that follicular fluid from small follicles (8–13 mm) shows distinctly different metabolic signatures compared to large follicles (17–22 mm), with differential expression of metabolites involved in steroid hormone biosynthesis [23]. Consequently, differences in collection protocols across studies may lead to non-comparable metabolic profiles.

In addition, pre-analytical factors such as sample handling, centrifugation, exposure to ambient conditions, and delays between collection and processing may affect metabolite stability. Storage conditions, including temperature, duration, and repeated freeze–thaw cycles, can lead to degradation or chemical transformation of metabolites, particularly lipids and redox-sensitive compounds, introducing systematic distortions in measured concentrations [24].

Furthermore, different analytical platforms capture fundamentally different portions of the metabolome, introducing inherent bias. No single analytical method can comprehensively detect all major chemical classes due to the chemical diversity of metabolites. Analytical platforms differ in sensitivity, resolution, and metabolite coverage. For example, NMR spectroscopy preferentially detects high-abundance metabolites, whereas mass spectrometry–based approaches allow the detection of low-abundance and structurally diverse compounds. These inherent differences may systematically bias the types of metabolites identified and pathway enrichment across studies, thereby affecting comparability and interpretation of results. The field lacks formal proficiency testing programs and standardized guidelines specific to follicular fluid metabolomics, making it essential that researchers fully document all preanalytical and analytical procedures to enable proper interpretation and cross-study comparisons [25, 26].

Regarding the present systematic review, although the protocol was retrospectively registered in the Open Science Framework (OSF), prospective registration would have reduced the risk of bias. Furthermore, the search strategy was restricted to three major databases and did not include gray literature or conference proceedings. Although these databases comprehensively cover biomedical literature, the exclusion of gray literature may have resulted in the omission of potentially relevant studies. In addition, the search terms were intentionally focused to ensure specificity; however, this approach may have limited sensitivity and contributed to the potential exclusion of studies using alternative terminology. Although studies published in both English and Portuguese were included, relevant studies in other languages may not have been captured. Another limitation of this review is that title and abstract screening was primarily conducted by a single reviewer. However, all excluded records were independently reassessed by a second reviewer, reducing the risk of erroneous exclusions. In addition, full-text articles were evaluated in duplicate. Although this approach strengthens the robustness of study selection, it does not fully correspond to complete duplicate screening at all stages. All these factors should be considered when interpreting the comprehensiveness of this review.

### Future perspectives

Currently, the application of metabolomics in reproductive medicine remains largely restricted to the research setting. Although several studies have demonstrated associations between metabolomic profiles and different infertility conditions, the clinical applicability of these findings is still limited. Further standardization of methodologies, validation in larger cohorts, and clarification of the biological significance of identified metabolites are required before routine clinical implementation can be considered.

Nevertheless, the available evidence suggests that metabolomics may represent a promising tool for improving the understanding of the follicular microenvironment and its relationship with oocyte competence [27]. In this context, future integration with computational approaches, including artificial intelligence, could potentially enhance predictive models of oocyte quality [28].

For instance, metabolomic analysis of follicular fluid may contribute to the development of non-invasive biomarkers to support oocyte selection. However, such applications require robust validation, particularly regarding their reproducibility, clinical utility, and cost-effectiveness, before being incorporated into assisted reproduction practice [29].

## Conclusion

This study evaluated how the follicular fluid metabolome varies across different female infertility conditions, both directly and indirectly. The findings demonstrate consistent alterations in metabolic profiles according to the underlying condition, highlighting the potential of metabolomics to identify clinically relevant biomarkers and support more individualized therapeutic approaches.

Metabolomic analysis emerges as a promising tool for improving both laboratory performance and clinical outcomes, particularly through its ability to characterize key pathways such as lipid metabolism, oxidative stress, and steroidogenesis. Although current evidence already allows the identification of metabolites most affected by infertility-related conditions, further advances in analytical accuracy and quantification are needed to fully translate these findings into clinical practice.

As the field evolves, metabolomics may enable the development of targeted interventions aimed at correcting metabolic imbalances within the follicular microenvironment, ultimately contributing to improved oocyte quality and higher chances of successful outcomes. In this context, the integration of metabolomics with emerging technologies, including artificial intelligence, has the potential to reshape clinical workflows and decision-making in assisted reproduction.

## Supplementary Information

Below is the link to the electronic supplementary material.

Supplementary file1 (DOCX 266 kb)

Supplementary file2 (DOCX 122 kb)

## Data Availability

This systematic review protocol was registered in the Open Science Framework (OSF)(https://osf.io/m74bg).
