# Supplementary material for: Metabolomic alterations in follicular fluid across female infertility conditions in IVF: a systematic review
Source: J Assist Reprod Genet. 2026 Jun 4;43(8):2321–39. doi: 10.1007/s10815-026-03925-y (PMC13597975; doi:10.1007/s10815-026-03925-y)
Supplement: Supplementary file 2 [file 10815_2026_3925_MOESM2_ESM.docx]

**Supplementary Information**

**Metabolomic alterations in follicular fluid across female infertility conditions in IVF: a systematic review**

| DATABASES: | Medline, Embase, Cochrane |
| --- | --- |
| SEARCH STRING: | ("Metabolomics") AND ("Follicular fluid") AND ("In vitro fertilization treatment") AND ("Human") |
| DELIMITATION: | PICO |
| FIRST SCREENING: | Rayyan tool |
| ARTICLES’ SELECTION: | Inclusion and exclusion criteria |
| ARTICLES’ REVIEW: | Authors’ doublecheck |
| DATA EXTRACTION: | Tables and figures |
| QUALITY ASSESSMENT: | Newcastle-ottawa score |

**Supplement 2** Summary of methodology used in the study.

**Supplement 3** Studies selected according to year of publication (A) and location of the study center (B).

| **Author** | **Year** | **Q1** | **Q2** | **Q3** | **Q4** | **Q5** | **Q6** | **Q7** | **Q8** | **TOTAL** |
| --- | --- | --- | --- | --- | --- | --- | --- | --- | --- | --- |
| **Xia, L.** | Sep-14 | 1 | 0 | 1 | 1 | 2 | 1 | 1 | 1 | 8 |
| **Cordeiro, F.** | Oct-15 | 1 | 0 | 1 | 1 | 2 | 1 | 1 | 1 | 8 |
| **Cordeiro, F.** | Mar-18 | 1 | 0 | 1 | 1 | 1 | 1 | 1 | 1 | 7 |
| **Karaer, A.** | Apr-18 | 1 | 0 | 1 | 1 | 1 | 1 | 1 | 1 | 7 |
| **Morelli, M.** | Jan-19 | 1 | 1 | 1 | 1 | 2 | 1 | 1 | 1 | 9 |
| **Song, J.** | Jul-19 | 1 | 1 | 0 | 1 | 1 | 1 | 1 | 1 | 7 |
| **Dogan, B.** | Jan-20 | 1 | 0 | 1 | 1 | 2 | 1 | 1 | 1 | 8 |
| **Zhang, X.** | May-20 | 1 | 1 | 1 | 1 | 2 | 1 | 1 | 1 | 9 |
| **Lazzarino, G.** | Aug-21 | 1 | 1 | 1 | 1 | 2 | 1 | 1 | 1 | 9 |
| **Guan, S.** | Sep-22 | 1 | 0 | 1 | 1 | 1 | 1 | 1 | 1 | 7 |
| **Shen, H.** | Oct-22 | 1 | 1 | 1 | 1 | 2 | 1 | 1 | 1 | 9 |
| **Dabaja,M** | Nov-22 | 1 | 0 | 1 | 1 | 2 | 1 | 1 | 1 | 8 |
| **Sun, Y.** | Jan-23 | 1 | 0 | 1 | 1 | 2 | 1 | 1 | 0 | 7 |
| **Li, J.** | Feb-23 | 1 | 0 | 1 | 1 | 1 | 1 | 1 | 1 | 7 |
| **Liu, A.** | Oct-23 | 1 | 0 | 1 | 1 | 2 | 1 | 1 | 1 | 8 |

**Supplement 4** Newcastle-Ottawa Scale for Methodological Quality Assessment
